# Death of Dr. L. P. Kennedy

**Published:** 1892-07

**Authors:** 


### DEATH OF DR. L. P. KENNEDY.

It is with pain and regret that we announce to the readers of THE JOURNAL that our former associate, Dr. Kennedy, died at his father's home in South Carolina, June 11th, in the thirty-second year of his age.

Dr. Kennedy was a graduate of Erskine College, Due West, S. C., and was afterwards elected tutor in the same institution, in which capacity he served most acceptably for four years. In order that he might perform well the duties of this new position soon after his election he repaired to Johns Hopkins University, and spent a year there in diligent study. He also attended the Summer School of Languages at Amherst.

Thus well equipped for his new position, he entered upon its duties, and no tutor ever gave greater satisfaction to his patrons, or was more successful in winning the love and confidence of his pupils.

His mind had for sometime been directed to the study of medicine, and with his accustomed energy and zeal, he applied himself to this new field under the direction of Dr. J. W. Wideman, of Due West.

In the fall of 1885, in company with a younger brother, he went to the University of New York, from which institution he was graduated in medicine, in March, 1887.

Soon after his graduation he received an appointment to one of the hospitals in New York; there, and in the hospitals of Brooklyn, he labored with great acceptance for one year and a half.

After his return to his native State he selected Atlanta for his future home, and there "a stranger in a strange city" he struggled and labored until the crown of victory was just within his grasp.

In many respects Dr. Kennedy was peculiarly qualified for the work of the physician. To his patients he was gentle, tender and sympathetic, and these have learned of his declining health and death with sincere regret. He was a gentleman of a noble christian character, and his actions and conduct were governed by the highest sense of honor. His sun went down while it was yet day, and thus ended a life that was exceptionally honorable and upright. His disease was that dread adversary which consumes its victim slowly but surely, and when the end came, he calmly accepted the issue "without fear and with a manly heart."

